# Great challenges in molecular medicine: toward personalized medicine

**DOI:** 10.3389/fcell.2013.00001

**Published:** 2013-10-04

**Authors:** Masaru Katoh

**Affiliations:** Katoh's Unit, National Cancer CenterTokyo, Japan

**Keywords:** bio-bank, database, knowledgebase, clinical sequencing, big data, WNT, FGF, Hedgehog

## Gene hunting, signaling network, and molecular medicine

**Figure 1:**
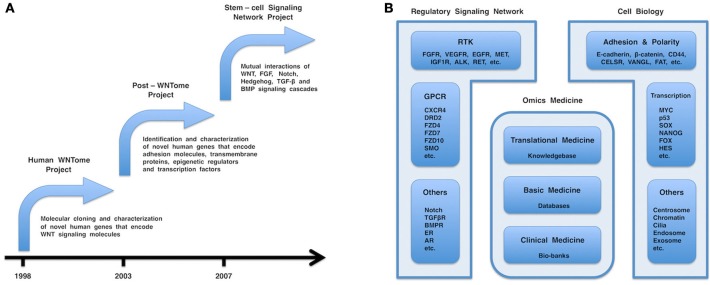
**(A)** Chronology of the human WNTome, post-WNTome, and stem-cell signaling network projects. **(B)** Fields in molecular medicine. Regulatory signaling network, cell biology, and omics medicine are the major fields in molecular medicine. Regulatory signaling network consists of receptor tyrosine kinase (RTK), G protein-coupled receptor (GPCR), and other signaling cascades, such as FGF, VEGF, WNT, Hedgehog, Notch, TGF-β, and BMP signaling cascades. Cell biology includes various important topics: cellular adhesion and polarity; centrosome biology; chromatin dynamics; endosome and exosome; and transcriptional regulation. Omics medicine consists of three layers: clinical medicine, followed by basic medicine and translational medicine. Bio-banks, databases, and a comprehensive knowledgebase are generated by clinical medicine, basic medicine, and translational medicine, respectively.

In the 20th century, most researchers investigated WNT signaling in cell and developmental biology by using model animals, such as *Drosophila, Xenopus*, and mouse. However, I was confident that WNT signaling should be investigated for clinical application by using human samples or cell lines. In 1998, I, together with post-doctoral fellows, started a human WNTome project to comprehensively clone and characterize novel human genes encoding WNT signaling molecules and to establish a “human WNT research” platform (Figure 1A). My group reported the molecular cloning and characterization of *FZD1, FZD3, FZD4, FZD6, FZD7, FZD8, FZD10, GIPC2, GIPC3, MFRP, NKD1, NKD2, VANGL1, WNT3A, WNT5B, WNT6, WNT7B, WNT8A, WNT9A (WNT14), WNT9B (WNT14B)*, and *WNT10A* as the major products of the human WNTome project [reviewed in Katoh (2002a)] and of other novel human genes, such as *FGF20, RhoU, RhoV*, and *SOX17*, as byproducts of the human WNTome project.

Most human genes that encode WNT signaling components had been cloned and characterized by 2002, whereas thousands of novel human genes outside of the WNT field still remained to be discovered. In 2003, colleagues and I started a post-WNTome project to identify and characterize novel human genes encoding adhesion molecules, transmembrane proteins, epigenetic regulators, and transcription factors (Figure 1A). My group reported *in silico* identification and characterization of novel human genes, such as *ANO1 (TMEM16A), ANO2 (TMEM16B), ANO3 (TMEM16C), ANO4 (TMEM16D), ANO5 (TMEM16E), ANO6 (TMEM16F), ANO7 (TMEM16G), ANO8 (TMEM16H), ASXL2, ASXL3, BCL9L, CDC50A (TMEM30A), CDC50B (TMEM30B), CDC50C (TMEM30C), CRB2, DACT1 (DAPPER1), DACT2 (DAPPER2), DIXDC1, FAT4, FMNL1, FMNL2, FMNL3, FOXR1 (FOXN5), FOXR2 (FOXN6), HES2, HES3, HES5, JMJD1C (TRIP8), JMJD2A (KDM4A), JMJD2B (KDM4B), JMJD2C (KDM4C), JMJD2D (KDM4D), KIF27, MPP7, PRICKLE1*, and *PRICKLE2*.

The human WNTome and post-WNTome projects were gene-hunting adventures that utilized molecular biology and computational biology, respectively. Inter- and intra-cellular signaling networks were simplified to a secondary picture consisting of nodes and edges. Nodes correspond to genes, mRNAs, proteins, or micro-RNAs (miRNAs), while edges correspond to their interactions. I then shifted my interest from the nodes to the edges and the whole picture. In 2007, my laboratory started a stem-cell signaling network project to elucidate mutual interactions of the WNT, FGF, Notch, Hedgehog, TGF-β, and BMP signaling cascades (Figure 1A) (Katoh and Katoh, 2007, 2009; Katoh and Nakagama, 2013).

Recently, I was appointed as the chief editor of Frontiers in Molecular Medicine, a sub- or specialty journal of Frontiers in Cell and Developmental Biology. I would like to contribute to the global scientific community through Frontiers in Molecular Medicine, which aims to address the gap between cell and developmental biology and clinical medicine and to promote development of novel diagnostics and therapeutics for a variety of human diseases, including cancers, cardiovascular diseases, diabetes mellitus, eye diseases, inflammatory bowel diseases, kidney diseases, liver diseases, neurological diseases, and respiratory diseases.

## Molecular medicine targeted to the regulatory signaling network

Mouse mammary tumor virus (MMTV) integrates at the *Wnt1, Wnt3, Wnt10b, Fgf3, Fgf4*, or *Fgf10* loci and induces mammary tumorigenesis [Dickson et al., 1984; Nusse et al., 1984; reviewed in Katoh (2002b)]. WNT signals are transduced through Frizzled (FZD) receptors and LRP5/6 or ROR1/2 co-receptors at both the canonical and non-canonical signaling cascades (Katoh and Katoh, 2007). Canonical WNT signals regulate cell fate (Clevers, 2006) in co-operation with FGF and Notch signals, while non-canonical WNT signals regulate cell morphogenesis and motility (Chien et al., 2009) in co-operation with FGF, TGF-β, and Hedgehog signals. The WNT, FGF, Notch, Hedgehog, and TGF-β signaling cascades constitute the stem cell signaling network, which orchestrates fetal development and post-natal homeostasis, whereas dysregulation of the stem cell signaling network causes a variety of hereditary and sporadic diseases (Katoh and Katoh, 2007).

Receptor tyrosine kinases (RTKs) are single-span transmembrane receptors with extracellular ligand-binding domain and intracelullar tyrosine kinase domain and are involved in growth factor signaling to downstream cascades, including the RAS-MAPK, PI3K-AKT, SRC, and PKC signaling cascades. FGFR1, FGFR2, FGFR3, and FGFR4 are receptors of the FGF family of ligands (Eswarakumar et al., 2005; Beenken and Mohammadi, 2009), and VEGFR1, VEGFR2, and VEGFR3 are receptors of the VEGF family of ligands (Tammela et al., 2005; Ellis and Hicklin, 2008). Growing evidence indicates that the WNT, Notch, Hedgehog, and TGF-β signaling cascades cross-talk with the FGFR signaling cascade, as well as other RTK signaling cascades. Because genetic alterations in RTKs, such as FGFRs, EGFR, HER2, MET, ALK, and RET, drive human cancers, small-molecule inhibitors and human/humanized monoclonal antibodies targeted against RTKs have been developed as cancer therapeutics (Ciardiello and Tortora, 2008; Kwak et al., 2010; Mologni, 2011; Buettner et al., 2013; Katoh and Nakagama, 2013; Li et al., 2013). VEGF antibodies are used as therapeutic agents for cancers and neovascular age-related macular degeneration (AMD) (Penn et al., 2008; Katoh, 2013b). Because clinical sequencing may reveal genetic changes underlining diseases without established therapeutics, the application of RTK inhibitors for previously unknown disease entities based on genetic alterations should be published as regular reports or case reports. Therapeutics targeting RTKs in cancers and non-cancerous diseases are important issues that will be published in Frontiers in Molecular Medicine.

G protein-coupled receptors (GPCRs) are seven transmembrane receptors that are linked to small G-protein signaling and other atypical signaling cascades. WNT receptors, including Frizzled-1 (FZD1), FZD2, FZD3, FZD4, FZD5, FZD6, FZD7, FZD8, FZD9, and FZD10, as well as Hedgehog signal transducer Smoothened, belong to the GPCR superfamily (Sagara et al., 1998; Koike et al., 1999; Katoh and Katoh, 2007; Lappano and Maggiolini, 2011). GPCRs are classified as class A (Rhodopsin family), class B (Secretin and Adhesion family), class C (Glutamate family), or class D (others, such as FZD/Smoothened and Taste2 family) (Lagerström and Schiöth, 2008). High-resolution structures of class A GPCRs have been reported, and those of class B GPCRs have also been recently reported (Sexton and Wootten, 2013). Small-molecule inhibitors targeted to Smoothened and monoclonal antibody targeted to FZD7 have been developed as cancer therapeutics (Katoh and Katoh, 2009; Philips et al., 2011; Arzumanyan et al., 2012; Gurney et al., 2012). Therapeutics targeting GPCRs in cancers and non-cancerous diseases are also important topics that will be published in Frontiers in Molecular Medicine.

The WNT, FGF, Notch, Hedgehog, and TGF-β signaling network is the tip of the iceberg for the regulatory signaling network that consists of signaling cascades via RTKs, GPCRs and other receptors (Figure 1B). Mutual interactions of this regulatory signaling network should be comprehensively investigated for the development and optimization of therapeutics.

## Molecular medicine targeted to cell biology

Cellular adhesion, cellular polarity, cellular proliferation, cellular survival, chromatin modification, cilia formation, DNA repair, endocytosis, exocytosis, and transcriptional regulation are all major topics in cell and developmental biology and molecular medicine (Figure 1B).

Forkhead-box (FOX) family members are DNA-binding proteins with a FOX domain that consists of two wing-like loops and three α-helices (Carlsson and Mahlapuu, 2002; Katoh and Katoh, 2004a; Hannenhalli and Kaestner, 2009). Because FOX proteins are involved in transcriptional regulation and DNA repair (Katoh et al., 2013), germ-line mutations in the *FOX* family of genes cause hereditary diseases, such as Axenfeld-Rieger syndrome; lymphedema-distichiasis syndrome; blepharophimosis, ptosis and epicanthus inversus syndrome; and speech and language disorder (Lehmann et al., 2003). Somatic mutations in the *FOX* family of genes, including gene amplifications, point mutations, and translocations, occur in a variety of human cancers (Katoh et al., 2013). My group identified and characterized the *FOXR1 (FOXN5)* gene within a cancer-associated deleted region in human chromosome 11q23.3 in 2004 (Katoh and Katoh, 2004c); Santo et al. later reported a FOXR1-MLL1 fusion caused by an intrachromosomal deletion in neuroblastoma in 2012 (Santo et al., 2012).

The *Drosophila Asx* gene encodes an epigenetic regulator that is associated with the Polycomb-group (PcG) repressor complex and the trithorax-group (trxG) activator complex (Jürgens, 1985; Sinclair et al., 1998; Brock and Fisher, 2005). ASXL1 (Fisher et al., 2003), ASXL2 (Katoh and Katoh, 2003), and ASXL3 (Katoh and Katoh, 2004b), which contain ASXN, ASXH, ASXM1, ASXM2, and PHD domains, are human homologs of the *Drosophila* Asx. BAP1, KDM1A (LSD1), NCOA1, the retinoic acid receptors (RARα and RARβ), estrogen receptor (ER), and glucocorticoid receptor (GR) are binding partners of ASXL1. Germ-line mutations in *ASXL1* occur in Bohring-Opitz syndrome, while somatic mutations in *ASXL1* occur in colorectal cancer with microsatellite instability, hematological malignancies and castration-resistant prostate cancer (Gelsi-Boyer et al., 2012; Katoh, 2013a and references therein).

Extracellular DNA and circulating miRNAs are key topics in translational medicine (Wittmann and Jäck, 2010; Turchinovich et al., 2012), and epigenetics play a key role in cancerous and non-cancerous diseases (Ordovás and Smith, 2010; Baylin and Jones, 2011). I recently underlined diagnostic techniques utilizing circulating miRNAs in exosomes and microsomes, therapeutics utilizing siRNAs in polymer-based hydrogel nanoparticles and therapeutics targeted to a field of epigenetic alterations (Katoh, 2013b). Manuscripts on a various aspects of cell and developmental biology that are applicable for molecular medicine are also welcome for publication in Frontiers in Molecular Medicine.

## The three-layer structure of omics medicine: bio-banks, databases, and a comprehensive knowledgebase

Genomics, transcriptomics, proteomics and metabolomics are representative “omics” disciplines of life science that deal with the entirety of genes, transcripts, proteins, and metabolites, respectively. Omics medicine is an emerging discipline of medical science that produces large amounts of omics data on genetics, genomics, epigenetics, transcriptomics, proteomics, and metabolomics. Here, I propose my personal view on the three-layer structure of omics medicine (Figure 1B). The first layer of omics medicine corresponds to clinical medicine that involves with patients' care and clinical sampling of blood and tissues (bio-banks). The second layer of omics medicine corresponds to basic medicine that produces cutting-edge data by using conventional molecular biology technologies, as well as high-throughput omics data using microarrays and next-generation sequencing technologies. The third layer of omics medicine corresponds to translational medicine, which develops novel diagnostics and therapeutics. Bioinformatics used to generate curated databases from high-throughput raw data by using algorithms (techint) is classified into the second layer, while bioinformatics used to generate a knowledgebase from manuscripts and curated databases using either human intelligence or a Watson-type supercomputer (humint) is classified into the third layer. I was engaged in clinical medicine as a physician from 1986 to 1990 and in basic medicine on WNT and FGF signaling cascades from 1990 to 2002 and have been engaged in translational medicine on the WNT, FGF, Hedgehog, Notch, TGF-β and BMP signaling cascades since 2003. The emergence of molecular biology evoked a great rotation from clinical medicine to basic medicine in the 20th century, while computer and internet technologies, an aging demography and governmental financial burdens have been promoting a rotation from basic medicine to translational medicine in the 21st century.

Industry also consists of three layers. The first layer of industry includes agriculture, forestry, fishery, and mining; the second layer of industry includes manufacturing and construction; the third layer of industry includes financial business, commerce, service, information, and health care. The Industrial Revolution caused a shift from the first layer of industry to the second layer of industry during the 18th and 19th centuries, while the Internet Revolution and Lehman Shock promoted the prevalence of commodity-assembly manufacturing to reduce personnel expenses and material costs, causing another rotation from the second layer of industry to the third layer of industry in the 21st century.

There are many analogies between the three-layer structure and development process of omics medicine and those of industry. Internet technology enabled the outsourcing of diagnostics and therapeutic optimization, which are known as telemedicine. Microarray and next-generation sequencing technologies concentrated the production of high-throughput data to world-class institutes or companies to reduce personnel and consumable expenses. Because the internet and high-throughput technologies are able to promote a leap from the first layer to the third layer of omics medicine, the global scientific community appears destined to move toward translational medicine.

Clinical medicine, basic medicine and translational medicine are responsible for the establishment and maintenance of bio-banks, databases and a comprehensive knowledgebase, respectively (Figure 1B). All of these aspects are mutually dependent and indispensable for clinical sequencing and molecular medicine in the era of personalized medicine. I am convinced that balanced support for clinical medicine, basic medicine and translational medicine are mandatory for the mechanistic elucidation of human diseases and the development of diagnostics and therapeutics.

## References

[B1] Arzumanyan A. Sambandam V. Clayton M. M. Choi S. S. Xie G. Diehl A. M.. (2012). Hedgehog signaling blockade delays hepatocarcinogenesis induced by hepatitis B virus X protein. Cancer Res. 72, 5912–5920. 10.1158/0008-5472.CAN-12-2329 22986746 PMC3521045

[B2] Baylin S. B. Jones P. A. (2011). A decade of exploring the cancer epigenome-biological and translational implications. Nat. Rev. Cancer 11, 726–734. 10.1038/nrc3130 21941284 PMC3307543

[B3] Beenken A. Mohammadi M. (2009). The FGF family: biology, pathophysiology and therapy. Nat. Rev. Drug Discov. 8, 235–253. 10.1038/nrd2792 19247306 PMC3684054

[B4] Brock H. W. Fisher C. L. (2005). Maintenance of gene expression patterns. Dev. Dyn. 232, 633–655. 10.1002/dvdy.20298 15704101

[B5] Buettner R. Wolf J. Thomas R. K. (2013). Lessons learned from lung cancer genomics: the emerging concept of individualized diagnostics and treatment. J. Clin. Oncol. 31, 1858–1865. 10.1200/JCO.2012.45.9867 23589544

[B6] Carlsson P. Mahlapuu M. (2002). Forkhead transcription factors: key players in development and metabolism. Dev. Biol. 250, 1–23. 10.1006/dbio.2002.0780 12297093

[B7] Chien A. J. Conrad W. H. Moon R. T. (2009). A Wnt survival guide: from flies to human disease. J. Invest. Dermatol. 129, 1614–1627. 10.1038/jid.2008.445 19177135 PMC3125088

[B8] Ciardiello F. Tortora G. (2008). EGFR antagonists in cancer treatment. N. Engl. J. Med. 358, 1160–1174. 10.1056/NEJMra0707704 18337605

[B9] Clevers H. (2006). Wnt/β-catenin signaling in development and disease. Cell 127, 469–480. 10.1016/j.cell.2006.10.018 17081971

[B10] Dickson C. Smith R. Brookes S. Peters G. (1984). Tumorigenesis by mouse mammary tumor virus: proviral activation of a cellular gene in the common integration region *int-2*. Cell 37, 529–536. 10.1016/0092-8674(84)90383-0 6327073

[B11] Ellis L. M. Hicklin D. J. (2008). VEGF-targeted therapy: mechanisms of anti-tumour activity. Nat. Rev. Cancer 8, 579–591. 10.1038/nrc2403 18596824

[B12] Eswarakumar V. P. Lax I. Schlessinger J. (2005). Cellular signaling by fibroblast growth factor receptors. Cytokine Growth Factor Rev. 16, 139–149. 10.1016/j.cytogfr.2005.01.001 15863030

[B13] Fisher C. L. Berger J. Randazzo F. Brock H. W. (2003). A human homolog of *Additional sex combs, ADDITIONAL SEX COMBS-LIKE 1*, maps to chromosome 20q11. Gene 306, 115–126. 10.1016/S0378-1119(03)00430-X 12657473

[B14] Gelsi-Boyer V. Brecqueville M. Devillier R. Murati A. Mozziconacci M. J. Birnbaum D. (2012). Mutations in ASXL1 are associated with poor prognosis across the spectrum of malignant myeloid diseases. J. Hematol. Oncol. 5, 12. 10.1186/1756-8722-5-12 22436456 PMC3355025

[B15] Gurney A. Axelrod F. Bond C. J. Cain J. Chartier C. Donigan L.. (2012). Wnt pathway inhibition via the targeting of Frizzled receptors results in decreased growth and tumorigenicity of human tumors. Proc. Natl. Acad. Sci. U.S.A. 109, 11717–11722. 10.1073/pnas.1120068109 22753465 PMC3406803

[B16] Hannenhalli S. Kaestner K. H. (2009). The evolution of *Fox* genes and their role in development and disease. Nat. Rev. Genet. 10, 233–240. 10.1038/nrg2523 19274050 PMC2733165

[B17] Jürgens G. (1985). A group of genes controlling the spatial expression of the bithorax complex in *Drosophila*. Nature 316, 153–155. 10.1038/316153a01350533

[B18] Katoh M. (2002a). Paradigm shift in gene-finding method: from bench-top approach to desk-top approach. Int. J. Mol. Med. 10, 677–682. 12429991

[B19] Katoh M. (2002b). *WNT* and *FGF* gene clusters. Int. J. Oncol. 21, 1269–1273. 12429977

[B20] Katoh M. (2013a). Functional and cancer genomics of ASXL family members. Br. J. Cancer 109, 299–306. 10.1038/bjc.2013.281 23736028 PMC3721406

[B21] Katoh M. (2013b). Therapeutics targeting angiogenesis: genetics and epigenetics, extracellular miRNAs and signaling networks. Int. J. Mol. Med. 32, 763–767. 10.3892/ijmm.2013.1444 23863927 PMC3812243

[B22] Katoh M. Igarashi M. Fukuda H. Nakagama H. Katoh M. (2013). Cancer genetics and genomics of human *FOX* family genes. Cancer Lett. 328, 198–206. 10.1016/j.canlet.2012.09.017 23022474

[B23] Katoh M. Katoh M. (2003). Identification and characterization of *ASXL2* gene *in silico*. Int. J. Oncol. 23, 845–850. 12888926

[B24] Katoh M. Katoh M. (2004a). Human *FOX* gene family. Int. J. Oncol. 25, 1495–500. 15492844

[B25] Katoh M. Katoh M. (2004b). Identification and characterization of ASXL3 gene in silico. Int. J. Oncol. 24, 1617–1622. 15138607

[B26] Katoh M. Katoh M. (2004c). Identification and characterization of human *FOXN5* and rat *Foxn5* genes *in silico*. Int. J. Oncol. 24, 1339–1344. 15067358

[B27] Katoh M. Katoh M. (2007). WNT signaling pathway and stem cell signaling network. Clin. Cancer Res. 13, 4042–4045. 10.1158/1078-0432.CCR-06-2316 17634527

[B28] Katoh Y. Katoh M. (2009). Hedgehog target genes: mechanisms of carcinogenesis induced by aberrant hedgehog signaling activation. Curr. Mol. Med. 9, 873–886. 10.2174/156652409789105570 19860666

[B29] Katoh M. Nakagama H. (2013). FGF receptors: cancer biology and therapeutics. Med. Res. Rev. [Epub ahead of print]. 10.1002/med.21288 23696246

[B30] Koike J. Takagi A. Miwa T. Hirai M. Terada M. Katoh M. (1999). Molecular cloning of *Frizzled-10*, a novel member of the *Frizzled* gene family. Biochem. Biophys. Res. Commun. 262, 39–43. 10.1006/bbrc.1999.1161 10448064

[B31] Kwak E. L. Bang Y. J. Camidge D. R. Shaw A. T. Solomon B. Maki R. G.. (2010). Anaplastic lymphoma kinase inhibition in non-small-cell lung cancer. N. Engl. J. Med. 363, 1693–1703. 10.1056/NEJMoa1006448 20979469 PMC3014291

[B32] Lagerström M. C. Schiöth H. B. (2008). Structural diversity of G protein-coupled receptors and significance for drug discovery. Nat. Rev. Drug Discov. 7, 339–357. 10.1038/nrd2518 18382464

[B33] Lappano R. Maggiolini M. (2011). G protein-coupled receptors: novel targets for drug discovery in cancer. Nat. Rev. Drug Discov. 10, 47–60. 10.1038/nrd3320 21193867

[B34] Lehmann O. J. Sowden J. C. Carlsson P. Jordan T. Bhattacharya S. S. (2003). Fox's in development and disease. Trends Genet. 19, 339–344. 10.1016/S0168-9525(03)00111-2 12801727

[B35] Li T. Kung H. J. Mack P. C. Gandara D. R. (2013). Genotyping and genomic profiling of non-small-cell lung cancer: implications for current and future therapies. J. Clin. Oncol. 31, 1039–1049. 10.1200/JCO.2012.45.3753 23401433 PMC3589700

[B36] Mologni L. (2011). Development of RET kinase inhibitors for targeted cancer therapy. Curr. Med. Chem. 18, 162–175. 10.2174/092986711794088308 21110809

[B37] Nusse R. van Ooyen A. Cox D. Fung Y. K. Varmus H. (1984). Mode of proviral activation of a putative mammary oncogene (*int-1*) on mouse chromosome 15. Nature 307, 131–136. 10.1038/307131a0 6318122

[B38] Ordovás J. M. Smith C. E. (2010). Epigenetics and cardiovascular disease. Nat. Rev. Cardiol. 7, 510–519. 10.1038/nrcardio.2010.104 20603647 PMC3075976

[B39] Penn J. S. Madan A. Caldwell R. B. Bartoli M. Caldwell R. W. Hartnett M. E. (2008). Vascular endothelial growth factor in eye disease. Prog. Retin. Eye Res. 27, 331–371. 10.1016/j.preteyeres.2008.05.001 18653375 PMC3682685

[B40] Philips G. M. Chan I. S. Swiderska M. Schroder V. T. Guy C. Karaca G. F.. (2011). Hedgehog signaling antagonist promotes regression of both liver fibrosis and hepatocellular carcinoma in a murine model of primary liver cancer. PLoS ONE 6:e23943. 10.1371/journal.pone.0023943 21912653 PMC3166282

[B41] Sagara N. Toda G. Hirai M. Terada M. Katoh M. (1998). Molecular cloning, differential expression, and chromosomal localization of human *Frizzled-1, Frizzled-2*, and *Frizzled-7*. Biochem. Biophys. Res. Commun. 252, 117–122. 10.1006/bbrc.1998.9607 9813155

[B42] Santo E. E. Ebus M. E. Koster J. Schulte J. H. Lakeman A. van Sluis P.. (2012). Oncogenic activation of *FOXR1* by 11q23 intrachromosomal deletion-fusions in neuroblastoma. Oncogene 31, 1571–1581. 10.1038/onc.2011.344 21860421

[B43] Sexton P. M. Wootten D. (2013). Structural biology: meet the B family. Nature 499, 417–418. 10.1038/nature12413 23863934

[B44] Sinclair D. A. Milne T. A. Hodgson J. W. Shellard J. Salinas C. A. Kyba M.. (1998). The *Additional sex combs* gene of *Drosophila* encodes a chromatin protein that binds to shared and unique Polycomb group sites on polytene chromosomes. Development 125, 1207–1216. 9477319 10.1242/dev.125.7.1207

[B45] Tammela T. Enholm B. Alitalo K. Paavonen K. (2005). The biology of vascular endothelial growth factors. Cardiovasc. Res. 65, 550–563. 10.1016/j.cardiores.2004.12.002 15664381

[B46] Turchinovich A. Weiz L. Burwinkel B. (2012). Extracellular miRNAs: the mystery of their origin and function. Trends Biochem. Sci. 37, 460–465. 10.1016/j.tibs.2012.08.003 22944280

[B47] Wittmann J. Jäck H. M. (2010). Serum microRNAs as powerful cancer biomarkers. Biochim. Biophys. Acta. 1806, 200–207. 20637263 10.1016/j.bbcan.2010.07.002

